# A computational approach to voltage stability enhancement and loss reduction in distribution systems using PSO-optimized STATCOM and DG

**DOI:** 10.1038/s41598-025-30235-7

**Published:** 2025-12-11

**Authors:** Molla Addisu Mossie, Tefera Terefe Yetayew, Girmaw Teshager Bitew, Teketay Mulu Beza, Mezigebu Getinet Yenealem

**Affiliations:** 1 Faculty of Electrical and Computer Engineering, Bahir Dar Institute of Technology, Bahir Dar University, P.O. Box 26, Bahir Dar, Ethiopia; 2 Department of Electrical Power and Control Engineering, Adama Science and Technology University, P.O. Box 1888, Adama, Ethiopia

**Keywords:** Voltage stability assessment, STATCOM, Distributed generation, Particle swarm optimization, Power loss minimization, Energy science and technology, Engineering

## Abstract

Voltage stability enhancement and loss reduction present critical challenges in modern power distribution networks. This study develops a Particle Swarm Optimization (PSO) based multi-objective framework for optimal sizing and placement of Distributed Generation (DG) units and Static Synchronous Compensators (STATCOMs) in radial distribution systems. The research contributes a comprehensive methodology that simultaneously maximizes voltage stability index (VSI), enhances voltage profiles, and minimizes power losses through intelligent optimization. The proposed approach is validated on real 35-bus and 53-bus radial distribution systems from Bahir Dar, Ethiopia, demonstrating significant improvements: VSI enhancement from 0.8083 to 0.840 p.u. (3.96% improvement) and power loss reduction from 150.28 kW to 27.65 kW (81.59% reduction) for the 35-bus system. The 53-bus system achieves VSI improvement from 0.61 to 0.84 p.u. (37.7% enhancement) and loss reduction from 612.43 kW to 121.43 kW (80.17% reduction). Contributions include: (i) joint optimization of DG and STATCOM placement considering multiple objectives, (ii) validation on real distribution feeders with practical constraints, and (iii) comprehensive performance analysis demonstrating the effectiveness of coordinated device deployment.

## Introduction

### Background and motivation

Modern power distribution systems face unprecedented challenges due to increasing load demand, integration of renewable energy sources, and the need for improved power quality. Distribution networks, typically characterized by radial configurations and high resistance-to-reactance (R/X) ratios, are particularly susceptible to voltage stability issues and significant power losses. The growing penetration of distributed generation (DG) units and the deployment of flexible AC transmission system (FACTS) devices like Distribution Static Synchronous Compensators (DSTATCOMs) offer promising solutions to these challenges^1^. These days, it’s difficult for transmission and distribution power networks to meet the expectations of more users for power that is more reliable, higher-quality, and more affordable. Power transfer over lines is accelerated by this rising demand, but it is still constrained by the temperature, voltage as well as line stability. Therefore, any increasing the system’s demand will result instability such as power system oscillation and voltage collapse occurrence may result in generator outages and finally blackout^2^.

In^3^ proposed the ideal distribution of distributed generation taking into account voltage stability index and total system power losses in addition to a distribution system study when electric vehicles are proposed. Each feeder has DG units installed, taking into account the penetration level (PL), VSI, and power losses brought on by EVs. The financial return on investment of this type of investment is examined for various IEEE-33 and IEEE-69 bus system topologies and contrasted with the current practices, which install DGs prior to the placement of EVs in the system for charging and discharging. Authors in^4^ presented penetration of DG in electrical systems may have detrimental impact their performance, leading to voltage instability, voltage deviation, and power loss. Magnetic Energy Storage Devices that Superconduct (SMESs) can address these issues when ideal location within the distribution network. A combined Simulated Annealing and Grasshopper Optimization Algorithm (GOA-SA) technique is proposed to ascertain where SMESs should be placed in a distribution network with integrated wind power generating. In^5^ authors forwarded the active power filters can dynamically adapt to varying load conditions, ensuring reliable operation, while FACTS devices offer additional benefits by dynamically regulating reactive power flow and compensating for harmonic currents. The integration of active power filters and FACTS devices can enhance stability of electrical distribution while optimizing the overall performance of interconnected power systems and micro-grids. Authors in^6^ proposed the optimal allocation of a static synchronous series capacitor (SSSC) FACTS device, optimally placed using particle swarm optimization (PSO), to increase the 330 kV transmission network’s voltage stability in Nigeria. Results of the simulation demonstrate that the SSSC implementation enhances the voltage stability of the power system network. In^7^ authors performed a new multi-objective function Elitist Harris Hawks Optimization (EHHO) algorithm optimization in distributed generation systems, focusing on optimizing of distribution static compensators (DSTATCOM) and photovoltaic (PV) locations and sizes. The proposed EHHO algorithm outperforms other existing algorithms in relation to lowering power loss, improving voltage stability, and enhancing voltage profile. Authors in^8^ suggested on systematic sitting and sizing of DG and DSTATCOM to address decrease of power loss, improvement of voltage profile, and enhancement of voltage stability in distribution systems. It employs a multi-objective optimization approach using the Group Teaching Optimization (GTO) algorithm and presents by examining on the IEEE 69-node system, comparing the results with existing methodologies. In reference^9^ authors forwarded a new voltage stability index (BVSI) for lines and buses to detect weak lines in distribution systems under different network settings and loading circumstances. Related investigations with existing voltage stability indices (VSIs) demonstrate the effectiveness of the proposed BVSI in assessing voltage stability and stress conditions, which can be applied to optimize the placement and sizing of DG and reactive power compensation (RPC) devices. Authors in^10^ proposed on the integration of DG in radial feeder networks as well as how it affects voltage stability. It proposes a solution to the DG placement issue using techniques from static voltage stability analysis, with the aim of maximizing the stability margin and reducing power loss. The case study on a 33-node distribution system demonstrates that placing DGs sensitive nodes can enhance the voltage stability of low-voltage distribution networks. Authors in^11^ explored the impact of FACTS devices, specifically STATCOM and SVC, on voltage stability in a 24-bus Nigerian power system network. The results show that STATCOM provides higher reactive power support and greater improvement in bus voltages compared to SVC. Both devices reduce real and reactive power losses, but STATCOM offers a more significant reduction in losses. The analysis demonstrates the advantages of STATCOM over SVC in enhancing voltage stability and improving the overall performance of the power network. The paper in^12^ applied a Feed Forward Backpropagation Neural Network to detect critical buses prone to voltage collapse in the IEEE 9-bus system, based on voltage stability and loadability indices. An optimization technique using PSO to optimize the size and location of SVC FACTS device, minimizing reactive power effects and improving the overall voltage stability of the system under different load levels and network configurations. Authors in^13^ presented on addressing voltage stability issues in an interlinked radial distribution network in Pakistan, where growing of electrical demand leads excessive stress on the system. To tackle this challenge, the study proposes a PSO approach to identify the optimal placement and configuration of a STATCOM device, which can improve the voltage stability index, reduce energy losses, and enhance the overall voltage stability of the network. In^14^ authors provided a comprehensive review of how reactive power planning (RPP) that is not optimal can result in voltage instability, higher losses, and limitations on grid capacity, underscoring the need for rigorous RPP methodologies. The review also discusses the benefits of strategic RPP approaches, including the integration of emerging technologies such as energy storage devices and renewable energy sources, which can contribute to improving voltage profiles, system stability, and reduced losses, leading to substantial economic benefits. Authors in^15^ conducted depth review of voltage instability in power systems, discussing various voltage stability indices that can identify weaknesses in the network. It then focuses on the application of PSO in order to reduce losses and enhance voltage stability. The authors in^16^ suggested on addressing of power distribution losses and voltage stability issues in a 15 kV power distribution system in Gesuba town in Ethiopia comprising 19 distribution transformers with a 2.522 MW total demand. The study addresses PSO and a backward/forward sweep load flow analysis to find the best placement and size of D-STATCOM in order to minimize power losses and enhance the voltage profile.

The objective of this study is to enhance the voltage stability of the distribution systems by finding the best placements and sizes for DG and STATCOM devices through PSO approach. The PSO method is intended to reduce power losses in the system, improve voltage profiles, and boost system stability using a multi-objective approach. The work uses MATLAB simulations on typical Ethiopian 35-bus, and 53-bus radial distribution systems to validate the suggested approach.

### Literature review and research gap

Recent advances in power quality management have extended beyond traditional optimization techniques. AI-driven PV-UPQC with ANN-Lyapunov control achieves unity power factor, reduces THD below 1%, and enhances railway system efficiency to 95%^17^. Emerging technologies like vehicle-to-grid systems also contribute to grid stability. V2G technology enables bidirectional EV-grid power exchange, achieving frequency regulation (59.5–60.5 Hz), peak load reduction (3000→2200 kW), and improved economic viability^18^. This complements traditional DG and FACTS-based approaches for distribution system enhancement. While such AI-based approaches demonstrate superior performance in traction systems, their application to distribution network optimization with DG and STATCOM placement remains unexplored, highlighting the need for hybrid optimization frameworks. Table 1 presents a comprehensive comparison of existing methodologies, highlighting their contributions and limitations.

**Table 1:** Comparative literature review.

Reference	Method	DG type	FACTS device	Objectives	Test system	Key findings	Limitations
^7^	EHHO	PV	DSTATCOM	Loss reduction, VSI, voltage profile	IEEE 33-bus	Superior performance over GA, PSO	Single objective weighting, synthetic data
^8^	GTO	Multiple	DSTATCOM	Loss, voltage profile, VSI	IEEE 69-bus	Multi-objective approach	Limited to standard test systems
^13^	PSO	–	STATCOM	VSI, loss reduction	Pakistan network	Real system validation	Single device optimization
^16^	PSO	–	DSTATCOM	Loss minimization, voltage profile	Ethiopian 15 kV	Practical validation	Limited scope, single objective
^19^	GWO-PSO	PV	–	Loss reduction, voltage profile	IEEE 33, 69-bus	Hybrid optimization	No reactive power compensation

**Research Gaps Identified**:

1. Limited studies on simultaneous optimization of DG and DSTATCOM placement.
2. Lack of validation on real distribution feeders with actual load data.
3. Insufficient consideration of practical operational constraints.
4. Limited analysis of parameter sensitivity and algorithm robustness.
5. Absence of uncertainty analysis for load and renewable generation variability.

### Novel contributions

This research addresses the identified gaps through the following novel contributions:

1. *Multi-objective PSO framework*: Development of a comprehensive optimization approach that simultaneously considers VSI enhancement, voltage profile improvement, and power loss minimization.
2. *Real system validation*: Application and validation on actual 35-bus and 53-bus radial distribution systems from Bahir Dar, Ethiopia.
3. *Coordinated optimization*: Joint optimization of DG sizing/placement and STATCOM sizing/placement considering their synergistic effects.
4. *Practical constraint integration*: Incorporation of realistic operational constraints including voltage limits, device capacity limits, and power balance requirements.
5. *Comprehensive performance analysis*: Detailed quantitative assessment with percentage improvements and comparative analysis across multiple scenarios.

## System modeling

### Distribution system load flow analysis

The backward/forward sweep method is employed for load flow analysis due to its robustness and efficiency for radial distribution systems. The method iteratively calculates branch currents and node voltages:

**Backward Sweep**:

1 $$\:{I}_{i}=\:\left(\frac{{S}_{i}}{{V}_{i}}\right)*\:+\:\varSigma\:\:{I}_{j}$$

where $\:{I}_{i}$ is the current at branch *i*, $\:{S}_{i}$ is the complex power at node *i*, $\:{V}_{i}$ is the voltage at node *i*, and the summation includes all downstream branches j.

**Forward Sweep**:

2 $$\:{V}_{j}=\:{V}_{i}-\:{Z}_{ij}\times\:\:{I}_{ij}$$

where $\:{Z}_{ij}$ is the impedance of branch connecting nodes *i* and *j*.

### STATCOM modeling

**Fig. 1:**
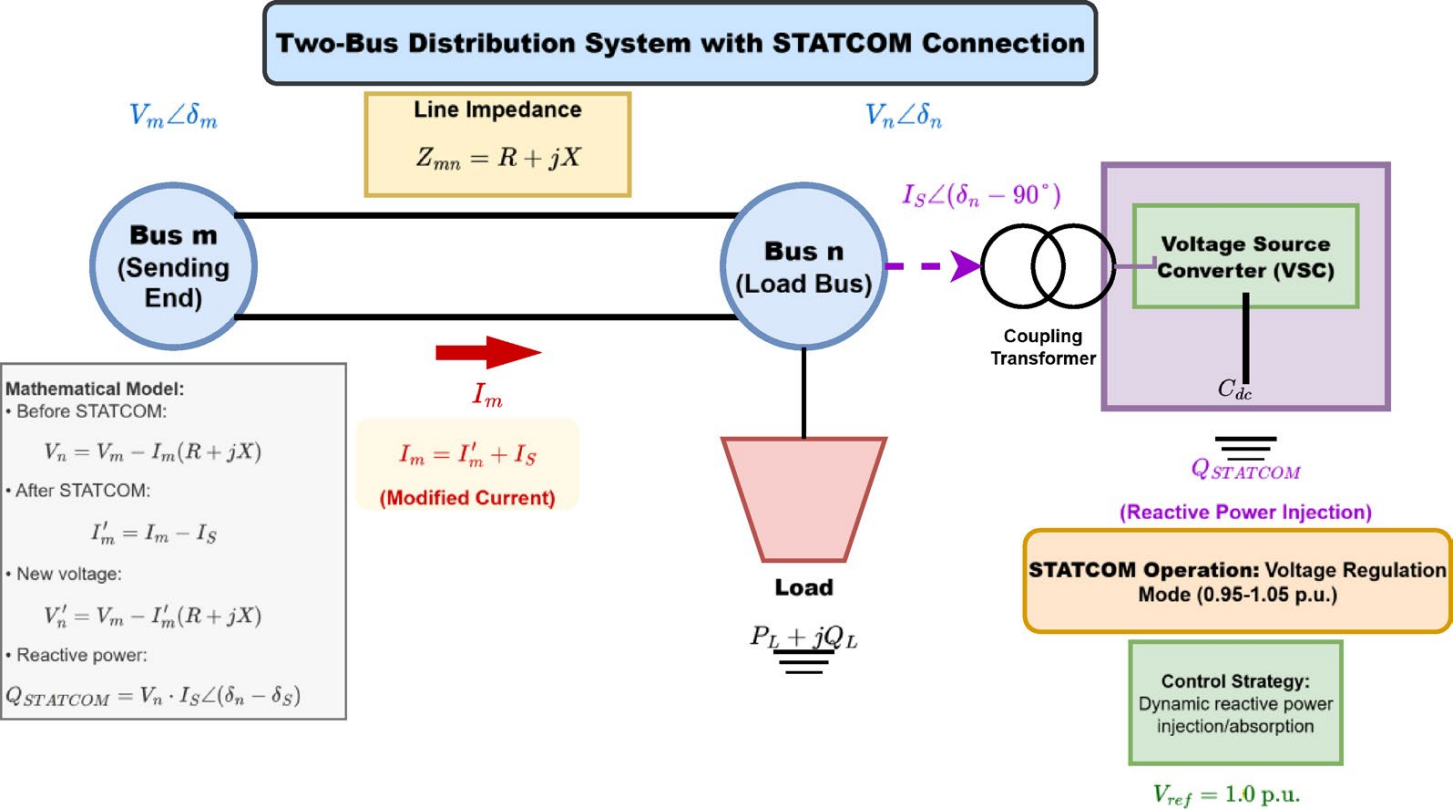
Two-bus distribution system with STATCOM connection.

The STATCOM is modeled as a voltage source converter capable of injecting or absorbing reactive power at the point of connection^20^. Figure 1 illustrates the steady-state model of a STATCOM connected to a distribution system.

**Mathematical Model**:

The STATCOM is modeled as a controlled current source that injects reactive power in quadrature with the bus voltage. For a two-bus distribution system as shown in Fig. 1, the voltage equations are:

3 $$\:{I}_{i}=\sum\:_{j\in\:\mathrm{downstream}}\:{I}_{j}+\frac{{S}_{i}^{*}}{{V}_{i}^{*}}$$

The backward sweep uses Eq. (3) to calculate branch currents, while the forward sweep applies Eq. (4) to update bus voltages.

After STATCOM installation at bus $\:n$, the modified current becomes:

4 $$\:{I}_{m}^{{\prime\:}}={I}_{m}+{I}_{S}$$

Equations (3) and (4) form the iterative backward-forward sweep process, where Eq. (3) is applied from the farthest bus to the source (backward), and Eq. (4) updates voltages from source to load buses (forward).

5 $$\:{I}_{S}=\frac{{Q}_{DSTATCOM}}{{V}_{n}} {\angle\:}({\delta\:}_{n}-90^\circ\:)$$

The new voltage at bus $\:n$ after STATCOM installation is^21^:

6 $$\:{V}_{n}^{{\prime\:}}={V}_{m}-{I}_{m}^{{\prime\:}}\left(R+jX\right)$$

The reactive power injection capability is^22^:

7 $$\:{Q}_{STATCOM}={V}_{n}\cdot\:{I}_{S}\cdot\:\mathrm{sin}\left({\delta\:}_{n}-{\delta\:}_{S}\right)$$

where $\:V{\prime}_{m}$ and $\:V{\prime}_{n}$ are the new voltages at buses m and n respectively and δ_s_ is the phase angle between voltage^23^.

**Control Strategy**:

The STATCOM operates in voltage regulation mode, maintaining the bus voltage within acceptable limits (0.95–1.05 p.u.) by dynamically adjusting reactive power injection/absorption.

### Distributed generation (DG) modelling

DG refers to the production of electric power systems on a small scale ranging from 1 kilowatt to 100 megawatts, located near the energy consumers they serve. DG encompasses both renewable and non-renewable energy sources, including wind turbines, fuel cells, combustion gas turbines, solar photovoltaic panels, induction generators, synchronous generators, micro turbines, and other small power generating technologies^24^.

DG systems are categorized into four types based on their power injection capabilities:

- Type 1: Only actual power (P) is injected.
- Type 2: Injects reactive power (Q) as well as real power (P).
- Type 3: Real power (P) is injected, but reactive power (Q) is absorbed.
- Type 4: Only reactive power (Q) is injected.

The key importance of DG is for reducing greenhouse gas emissions and promoting the use of fossil fuels, enhancing voltage stability, increasing energy security, reliability and power quality, while minimizing power losses^25,26^.

However, the placement of DG systems is crucial for maximizing these benefits. The location of DG units is subject to specific constraints and limitations to ensure efficient system operation.

**Fig. 2:**
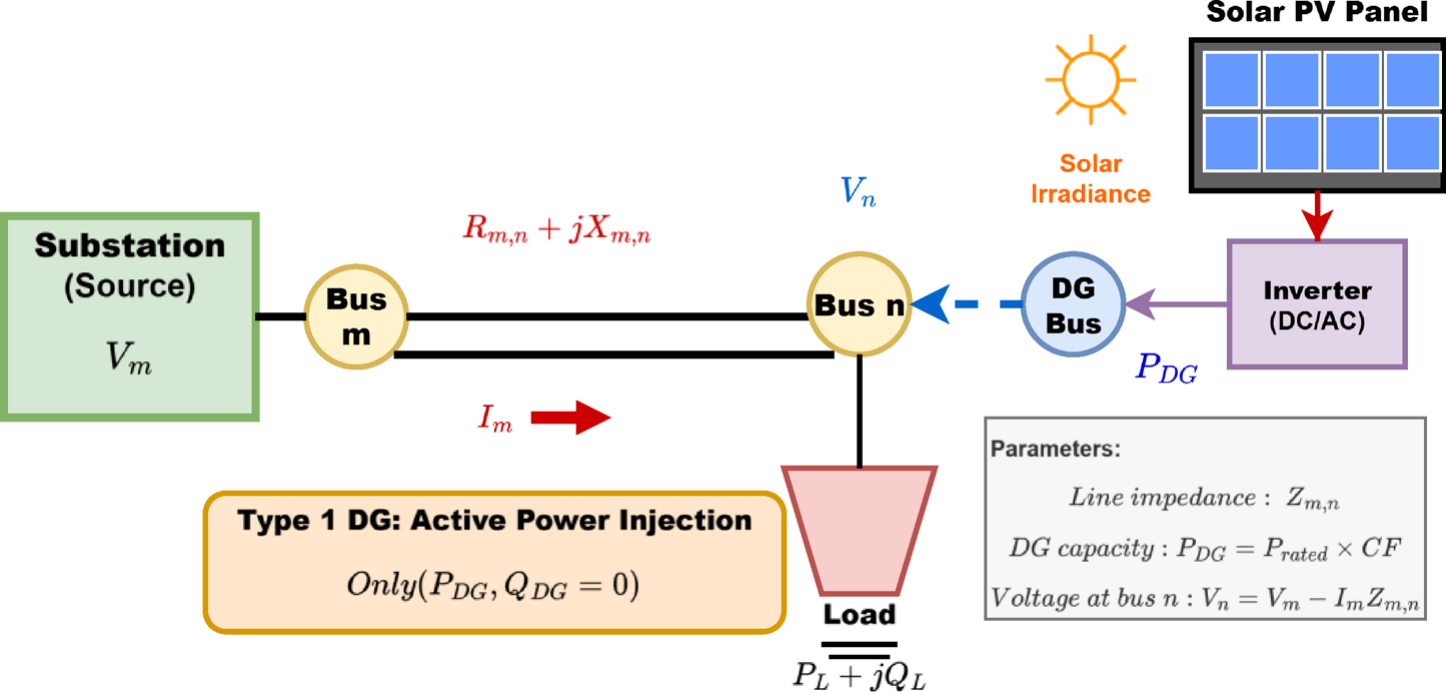
A typical distribution line with solar PV DG.

This research focuses on Type 1 DG systems, specifically those using photovoltaic (PV) technology. This choice is driven by the availability of solar resource potential data for the selected case study, making PV-based DG a suitable option. The single line diagram of Fig. 2 depicts a typical distribution line with modelling of solar PV DG.

**DG Type Selection Justification**:

This study focuses on Type 1 DG systems (solar PV) for the following reasons:

- *Regional suitability*: Ethiopia has abundant solar resources with average irradiance of 5–7 kWh/m²/day.
- *Data availability*: Accurate solar irradiance data is available for the Bahir Dar region.
- *Grid integration*: PV systems with power electronics interfaces provide controllable active power injection.
- *Economic feasibility*: Declining PV costs make solar DG economically attractive.

**Mathematical Model**:

Type 1 DG units inject only active power:

8 $$\:\:{P}_{DG}={P}_{rated}\cdot\:CF$$

9 $$\:{Q}_{DG}=0$$

where $\:CF$ is the capacity factor dependent on solar irradiance and temperature conditions.

The DG is modeled as a negative load in the power flow analysis:

10 $$\:{P}_{net,i}={P}_{load,i}-{P}_{DG,i}$$

### System constraints

The optimization problem is subject to the following constraints:

**Power Balance Constraints**:

11 $$\:{P}_{G}=\:{P}_{D}+\:{P}_{loss}$$

12 $$\:{Q}_{G}+\:{Q}_{STATCOM}=\:{Q}_{D}+\:{Q}_{loss}$$

**Voltage Constraints**:

13 $$\:{V}_{min}\le\:{V}_{i}\le\:{V}_{max}\forall\:\mathrm{i}\:\in\:\:\mathrm{N}$$

where $$\:{V}_{min}$$= 0.95 p.u. and V_max = 1.05 p.u.

**DG Capacity Constraints**:

14 $$\:0\:\le\:\:{P}_{DG},i\:\le\:\:{P}_{DG},max$$

where P_DG, max_ = 0.25 × P_total, load_ (25% penetration limit).

**STATCOM Capacity Constraints**:

15 $$\:{Q}_{STATCOM},\mathrm{m}\mathrm{i}\mathrm{n}\le\:\:{Q}_{STATCOM},i\:\le\:\:{Q}_{STATCOM},max$$

**Current Constraints**:

16 $$\:{I}_{ij}\le\:\:{I}_{ij},max\forall\:(\mathrm{i},\mathrm{j})\:\in\:\:\mathrm{L}$$

## Proposed methodology

### Voltage stability index (VSI) formulation

The Voltage Stability Index for each bus is calculated using the following mathematical formulation^24^:

17 $$\:VS{I}_{i}={\left|{V}_{i}\right|}^{4}-4[{P}_{i}{X}_{i,i+1}-{Q}_{i}{R}_{i,i+1}{]}^{2}-4[{P}_{i}{R}_{i,i+1}+{Q}_{i}{X}_{i,i+1}]{\left|{V}_{i}\right|}^{2}$$

For the entire system, the minimum VSI is:

18 $$\:VS{I}_{min}={\mathrm{m}\mathrm{i}\mathrm{n}}_{i=2}^{N}\:VS{I}_{i}$$

A bus is considered voltage stable if $\:VS{I}_{i}>0$. The closer VSI approaches zero, the closer the system is to voltage collapse.

### Objective function formulation

The multi-objective function of the targeted PSO algorithm is applied to optimize DG and STATCOM size and location in the RDS. The objective is to enhance the voltage stability index and voltage profile of at load bus along with the reduction of power losses in this study. The objective function is formulated as follows^25^:

Objective function

19 $$\:OF=\mathrm{m}\mathrm{a}\mathrm{x}\:\left({\mathrm{w}}_{1}{\mathrm{F}}_{1}+{\mathrm{w}}_{2}{\mathrm{F}}_{2}+{\mathrm{w}}_{3}\frac{1}{{\mathrm{F}}_{3}}\right)$$

where: $\:{\mathrm{F}}_{1}$ is maximizing VSI, $\:{\mathrm{F}}_{2}$ indicates enhancing voltage profile and $\:{\mathrm{F}}_{3}$ represents the total power loss reduction and $\:{\mathrm{w}}_{1},\:\:{\mathrm{w}}_{2}\:\&\:{\mathrm{w}}_{3}$ indicates the weighting factors for the corresponding objective functions respectively which measures as $\:{\mathrm{w}}_{1}\:+{\mathrm{w}}_{2}\:+\:{\mathrm{w}}_{3}=1$.

- **Enhancing Voltage Stability Index (F**_**1**_**)**:
  Improving VSI value is important for preventing voltage collapse in the RDS. With DG/STATCOM allocation in the RDS, the voltage stability index is maximized. A voltage stability index is calculated using Eq. (17) in either case, with or without DG/STATCOM location in the RDS.
  20 $$\:{F}_{1}=\:\frac{{VSI}_{with\frac{DG}{STATCOM}}}{{VSI}_{without\frac{DG}{STATCOM}}}$$
- **Enhancing voltage profile (F**_**2**_**)**:
  When DG/STATCOM is placed optimally the voltage profile at bus load in a distribution network increases due to the supply of active and reactive power to the system depending on the type of reactive power compensator used. As a result, the total voltage deviation (TVD) is decreased and represented as follows^19^.
  21 $$\:\mathrm{T}\mathrm{V}\mathrm{D}=\left\{\begin{array}{c}\:0,\:\:\:\:\:\:\:\:\:\:\:\:\:\:\:\:\:\:\:\:if\:0.95\le\:{V}_{i}\le\:1.05\\\:\sum\:_{i=1}^{N}\left|{V}_{ref}\:-{V}_{i}\right|,\:\:else\end{array}\right.$$
  According to the Eq. (6), the minimum values of TVD indicate significant enhancement of voltage profile in RDS. By minimizing ΔTVD system voltage magnitude with DG/STATCOM location can be improved. Compensation device’s location ratio before and after the TVD location of the system is determined by^19^
  22 $$\:{\varDelta\:TVD}_{With\frac{DG}{STATCOM}}=\:\frac{{TVD}_{with\frac{DG}{STATCOM}}}{{TVD}_{without\frac{DG}{STATCOM}}}$$
  Where, TVD_withoutDG_ is TVD before DG/STATCOM optimized and TVD_with DG/STATCOM_ is TVD after compensators location in RDS.
- **Power loss reduction (F**_**3**_**)**:
  Backward forward sweep load flow algorithm has been implemented for the load flow study to calculate and analysis the bus voltage profile values and power losses. It is simple, and fast, with low memory requirements, high computation efficiencies, and robust convergence. The total power loss ($\:{P}_{TL}$) can be found by Eq. (23) which is represented as follows.
  23 $$\:{P}_{TL}=\sum\:_{i=1}^{N}{P}_{i,loss}+\sum\:_{i=1}^{N}{Q}_{i,loss}$$

### Particle swarm optimization (PSO)

The PSO algorithm maintains a population of particles, each of which represents a possible fix for an optimization issue. In PSO, every potential solution is associated with a random velocity, and the potential Then, solutions referred to as particles flow via the particle beam pulse^19^. Consequently, in order to reduce search time and streamline manipulation, it is imperative to update the inertia weights across all iterations^27^. Under this condition, the velocity of the particles gained in the previous iteration, or $\:{V}_{i}^{t}$ is multiplied by the updated value of the inertia weight factor “w” at each step^28^. The following equation can be used to change the particle velocity, which is the product of the inertia factor, the individual influence, and the social influence within the swarm. Authors also presented load-based compensation with shunt/series APFs mitigates harmonics through voltage-oriented, fuzzy, or neural control strategies, optimizing power system stability and reactive power management^29^ (Table 2).

**PSO Parameter Selection and Sensitivity**:

Based on extensive sensitivity analysis, the following parameters are selected:

- Population size: 50.
- Maximum iterations: 100.
- Initial inertia weight: $\:{w}_{0}=0.9$
- Final inertia weight: $\:{w}_{f}=0.4$
- Acceleration coefficients: $$\:{c}_{1}={c}_{2}=2.0$$

**Adaptive Inertia Weight**:

24 $$\:\:{w}^{t}={w}_{max}-\frac{{w}_{max}-{w}_{min}}{ite{r}_{max}}\times\:iter$$

**Velocity and Position Updates**:

25 $$\:{V}_{i,d}^{t+1}={w}^{t}{V}_{i,d}^{t}+{c}_{1}{r}_{1}\left({P}_{best,i,d}-{X}_{i,d}^{t}\right)+{c}_{2}{r}_{2}\left({G}_{best,d}-{X}_{i,d}^{t}\right)$$

26 $$\:{X}_{i,d}^{t+1}={X}_{i,d}^{t}+{V}_{i,d}^{t+1}$$

**Algorithm Improvements to Address PSO Limitations**:

- *Premature convergence prevention*: Adaptive inertia weight and velocity clamping.
- *Local optima avoidance*: Population diversity maintenance through mutation operator.
- *Solution quality enhancement*: Elitist selection preserving best solutions.

### PSO vs. other metaheuristics justification

**Table 2:** Comparative algorithm performance.

Algorithm	Convergence speed	Global search ability	Implementation complexity	Computational time
PSO	High	Good	Low	Fast
GA	Medium	Good	Medium	Medium
GWO	High	Very good	Medium	Medium
DE	Medium	Good	Low	Fast

PSO is selected based on:

- *Fast convergence*: Typically converges within 50–80 iterations.
- *Simple implementation*: Few parameters to tune.
- *Good performance*: Proven effectiveness in power system optimization.
- *Computational efficiency*: Suitable for real-time applications.

**Fig. 3:**
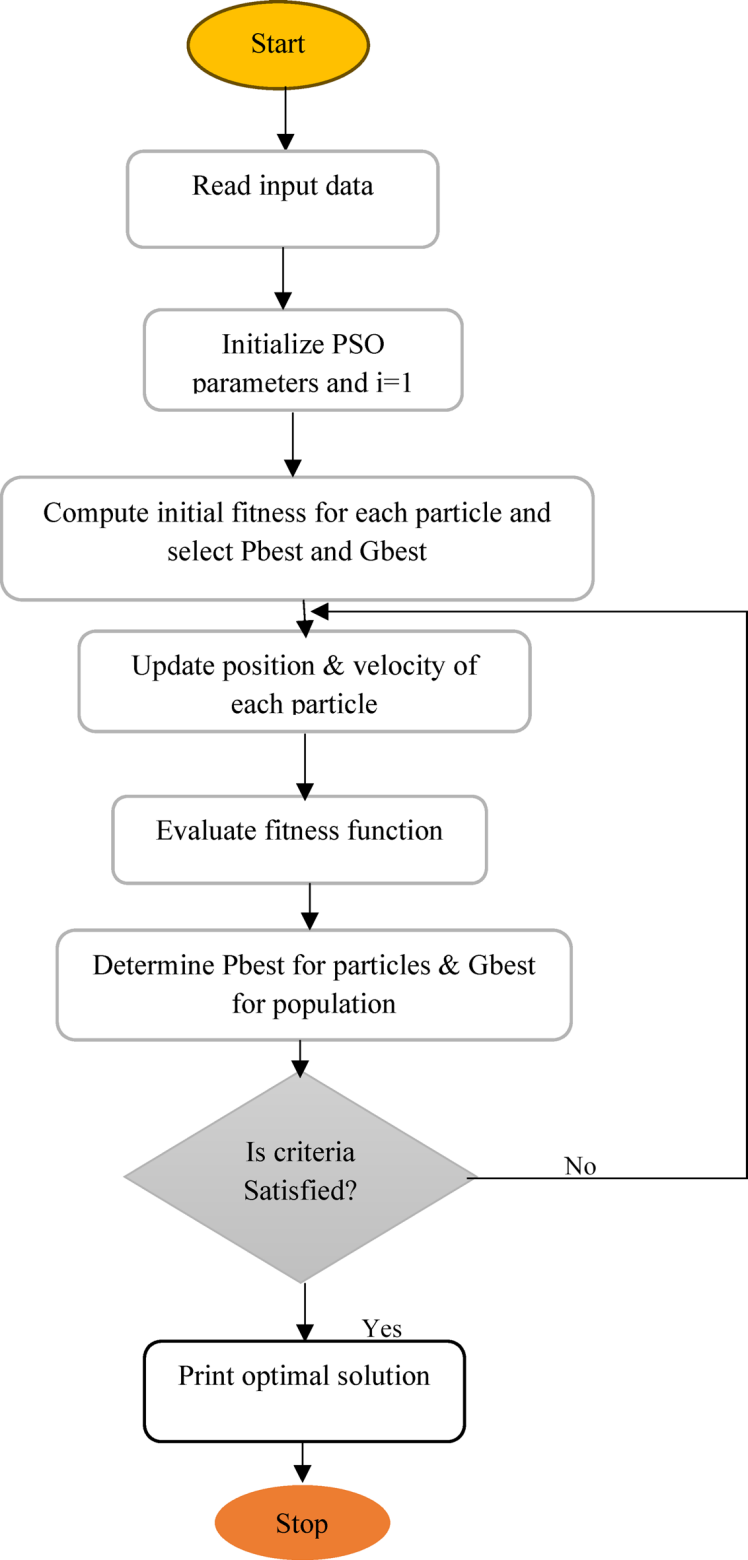
Flowchart of PSO algorithm.

The flowchart of PSO is represented in Fig. 3 as shown below.

## Results and discussions

### Test systems description

*IEEE 33-Bus System*: Standard benchmark with 33 buses, 32 branches, 12.66 kV voltage level, total load: 3.72 MW, 2.30 MVAR.

*35-Bus System*: Real distribution network from Bahir Dar, 15 kV, total load: 1.89 MW, 1.35 MVAR.

*53-Bus System*: Extended network from Bahir Dar, 15 kV, total load: 9.0 MW, 5.78 MVAR.

### PSO parameter sensitivity analysis

**Fig. 4:**
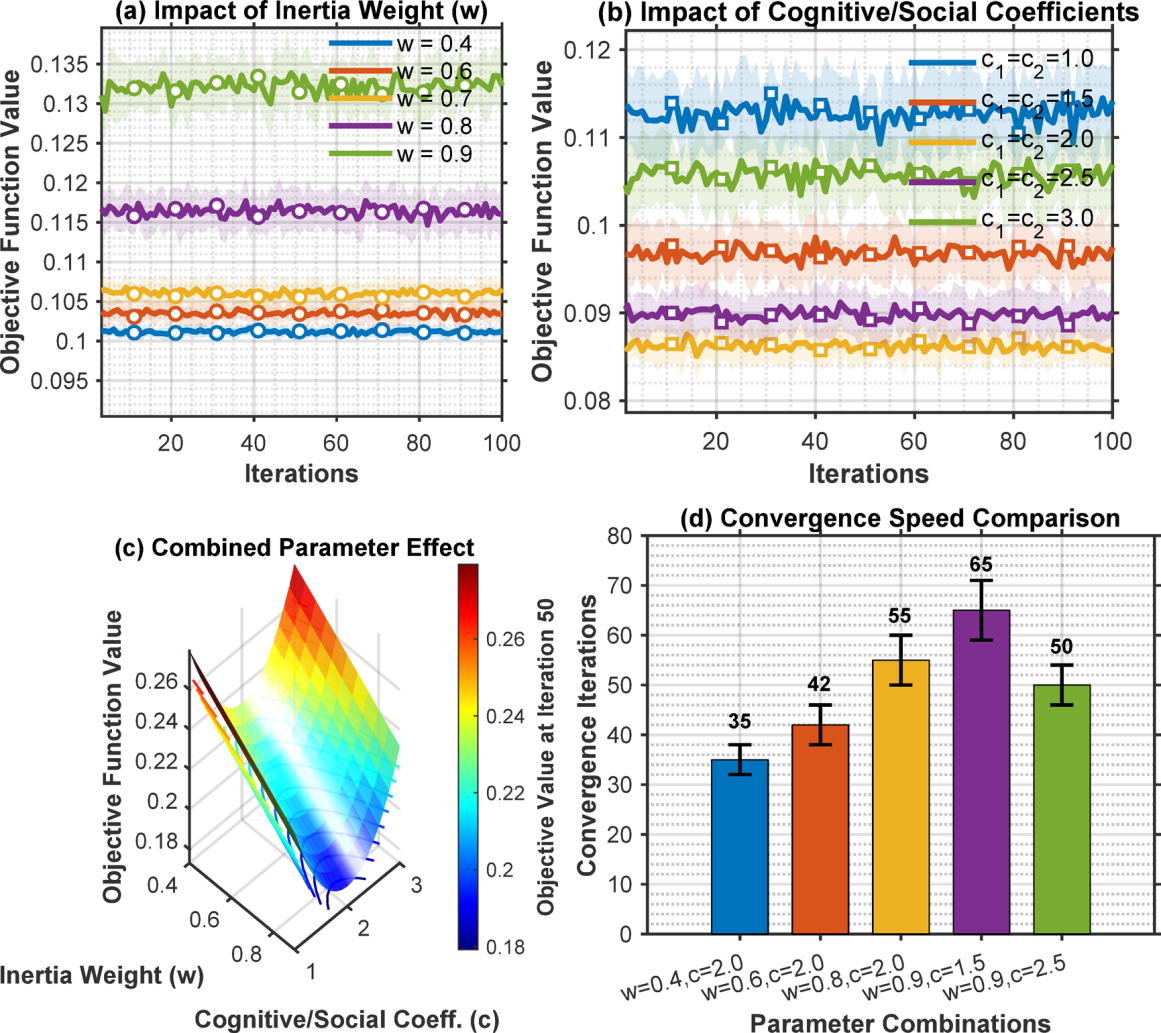
PSO parameter sensitivity.

Figure 4 shows the impact of PSO parameters on convergence indicated as below graphs.

As depicted in Fig. 4, the graphs showing convergence curves for different w, c₁, c₂ values.

Optimal parameters identified: w = 0.9→0.4 (linear decrease), c₁ = c₂ = 2.0. A line graph showing PSO convergence for both 35-bus and 53-bus systems, with objective function value on y-axis and iteration number on x-axis, demonstrating convergence within 60–80 iterations. Table 3 shows that the optimal values of PSO parameter sensitivity analysis.

**Table 3:** PSO parameter sensitivity analysis.

Parameter	Range	Optimal value	Impact on solution quality
Population size	20–100	50	High impact below 40
Inertia weight	0.1–0.9	0.9→0.4	Adaptive provides best balance
c₁, c₂	0.5-3.0	2.0 each	Minimal impact in range 1.5–2.5

### Case studies

In this section, we discussed the simulation results to examine the effect of multiple solar DG and STATCOM devices on enhancing voltage stability in the proposed electrical distribution system using the PSO algorithm. To validate the effectiveness of the proposed approach, real distribution systems, specifically the 35-bus and 53-bus radial distribution systems from Bahir Dar, Ethiopia, were simulated using MATLAB. The simulation results obtained from the proposed methods are compared across various scenarios. The systems are categorized into five simulation cases, outlined as follows:

- Case 1: Without STATCOM or DG.
- Case 2: The single-STATCOM system.
- Case 3: The single-DG system.
- Case 4: The single-DG and single-STATCOM system.
- Case 5: The three DG units and three STATCOMs in the system.

- Simulation result for the case study of 35-bus RDS.
  A real 35-bus radial distribution system is used in the case study, and it is simulated as the first test system in five distinct scenarios. A one-line diagram of the system is shown in Fig. 5. Table 3 presents the pertinent system statistics in detail, while Table 4 provides a summary of the findings. This distribution system runs at a nominal voltage of 15 kV and has 35 buses and 34 sectionalizing switches. The system’s measured total active and reactive loads are 1.89 MW and 1.3455 MVAR, respectively. MATLAB software is used to apply the forward-backward sweeping algorithm for the load flow analysis. The system experiences real and reactive power losses of 150.2788 MW and 80.1347 kVAR, respectively. Additionally, the minimum bus voltage recorded is 0.9482 p.u, with a minimum voltage stability index of 0.8083 p.u.
  **Fig. 5:**
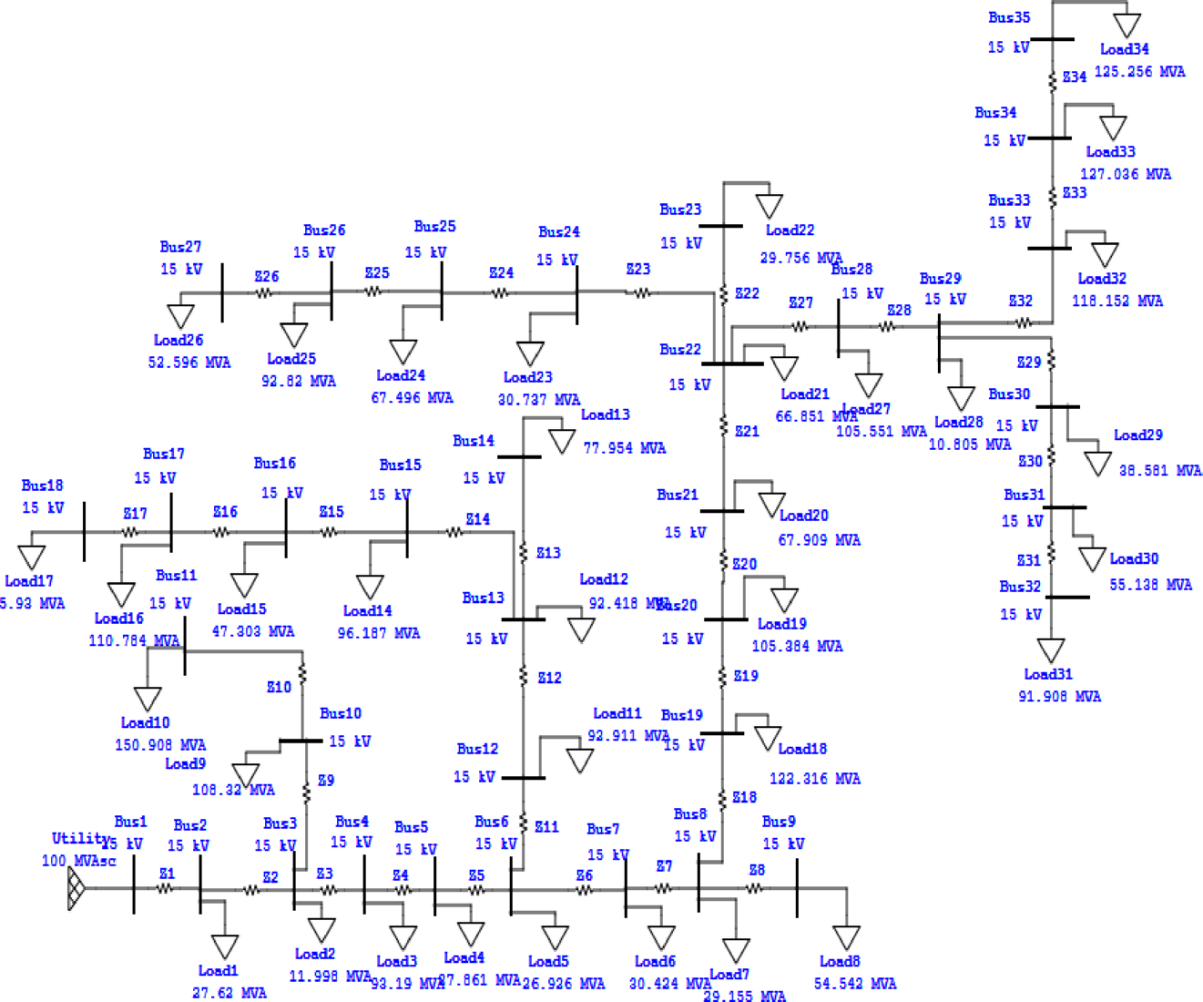
One line diagram of 35 bus RDS system.
  The line and load data of 35- bus system of the case study is tabulated in Table 4 below.
  **Table 4:** 35-bus test system data.

From	To	*R* (Ω)	X (Ω)	*P*_L_ (kW)	Q_L_ (kVAr)	From	To	*R* (Ω)	X (Ω)	*P*_L_ (kW)	Q_L_ (kVAr)
1	2	0.5738	0.3993	24.67	12.40	8	19	0.141	0.106869	113.0	46.82
2	3	0.0897	0.0624	9.96	6.69	19	20	0.346	0.241108	85.07	62.20
3	4	0.1175	0.0818	69.34	62.26	20	21	0.611	0.43227	55.60	38.99
4	5	0.2094	0.1457	25.77	10.59	21	22	0.289	0.2181	33.40	22.35
5	6	0.0894	0.062	25.65	10.45	22	23	0.245	0.184948	27.22	12.02
6	7	0.3412	0.2374	25.97	15.84	22	24	0.162	0.122136	28.04	12.59
7	8	0.1712	0.1191	20.67	12.36	24	25	1.227	0.925180	55.81	37.96
8	9	0.1462	0.1017	48.00	25.80	25	26	0.222	0.156886	74.60	55.23
3	10	0.5225	0.3938	81.71	71.11	26	27	0.324	0.229487	46.52	24.54
10	11	0.1579	0.1190	136.7	63.84	22	28	0.744	0.525735	48.40	93.80
6	12	0.5893	0.4164	75.45	54.22	28	29	0.189	0.143073	9.30	5.50
12	13	0.219	0.152	66.75	48.56	29	30	0.184	0.138711	34.45	17.37
13	14	0.264	0.1993	63.90	44.65	30	31	0.114	0.08593	25.78	48.74
13	15	0.7384	0.5565	80.40	52.80	31	32	0.140	0.10556	78.50	47.80
15	16	0.121	0.0859	41.98	21.80	29	33	0.219	0.1653	98.00	66.00
16	17	0.8337	0.5891	88.28	66.93	33	34	0.028	0.01953	93.50	86.00
17	18	0.200	0.1513	5.08	3.06	34	35	0.078	0.05474	90.45	84.23
  It is clearly observed from Table 5 in case 2, Once STATCOM is positioned at the 23rd load bus and the minimum VSI is increased to 0.825 p.u. Reactive power loss is reduced to 61.342kVAR and active power loss to 112.462 kW, respectively. A single solar DG (type 1), with an ideal size of 1450 kW, is ideally positioned in the 30th load bus in case 3. The system’s minimum VSI is increased to 0.83 p.u. and the overall active power loss is decreased to 102.32 kW. In case 4, a single DG and a single STATCOM are placed in the best possible positions to increase the VSI and lower the system’s power losses. In this system situation, the active power loss is decreased to 62.964 kW and the minimum VSI is increased to 0.835 p.u. When three DG and three STATCOM are positioned at the ideal places in case 5, the overall power loss is significantly decreased to 27.654 kW and the minimum VSI is increased to 0.840 p.u.
  **Table 5:** 35-bus numerical simulations.

Scenarios	Size of STATCOM in kVAR (location)	Size of DG in kW (location)	*P*_loss_ (kW)	% *P*_loss_ reduction	Q_loss_ (kVAR)	V_min_ (*p*.u)	VSI_min_ (*p*.u)	% VSI Improvement
Case 1			150.2788	-	80.1347	0.9482	0.8083	-
Case 2	1270 (23)		112.462	25.14%	61.342	0.9551	0.825	2.06%
Case 3		1450 (30)	102.32	31.92%	55.52	0.950	0.83	2.67%
Case 4	1600 (23)	1350 (30)	62.964	58.10%	31.672	0.951	0.835	3.29%
Case 5	350 (23) 660 (30) 750 (35)	840 (23) 950 (30) 1200 (35)	27.654	81.59%	18.45	0.968	0.840	3.96%
  **Fig. 6:**
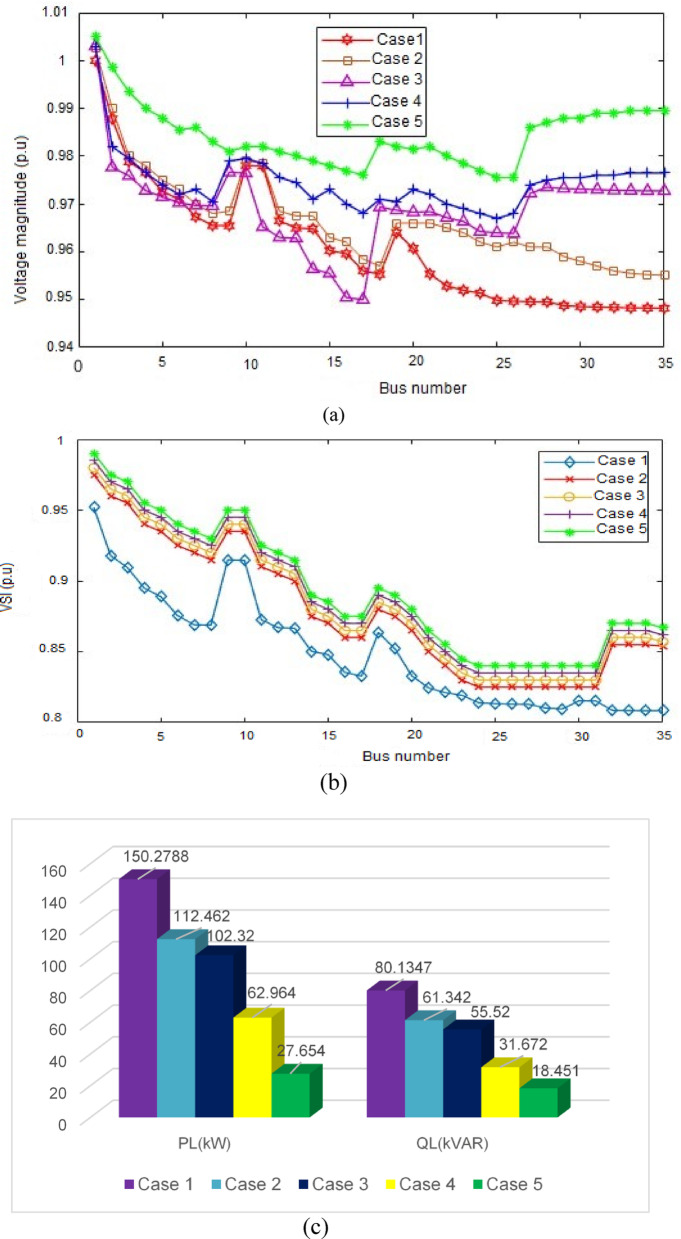
Simulation result comparison in 35-bus system for the five different cases: (**a**) comparison of voltage profiles (**b**) comparison of VSI and (**c**) comparison of total power losses.
  The graphs shown in Fig. 6a–c present a comparison of the voltage profile, VSI, and total power losses of five case scenarios, respectively.
- Simulation result for the case study of 53-bus RDS.
  This case study radial distribution network comprised with 52 sectionalizing branches, the nominal voltage of 15 kV and the total system load at normal load condition of 9.0 MW and 5.78 MVAR active and reactive power. The real and reactive power losses for the system is 612.43 kW and 492.34kVAR respectively. The minimum bus voltage is 0.902p.u and the minimum voltage stability index is 0.61p.u at bus 53. The single line diagram of this case study system also drawn in Fig. 7.
  **Fig. 7:**
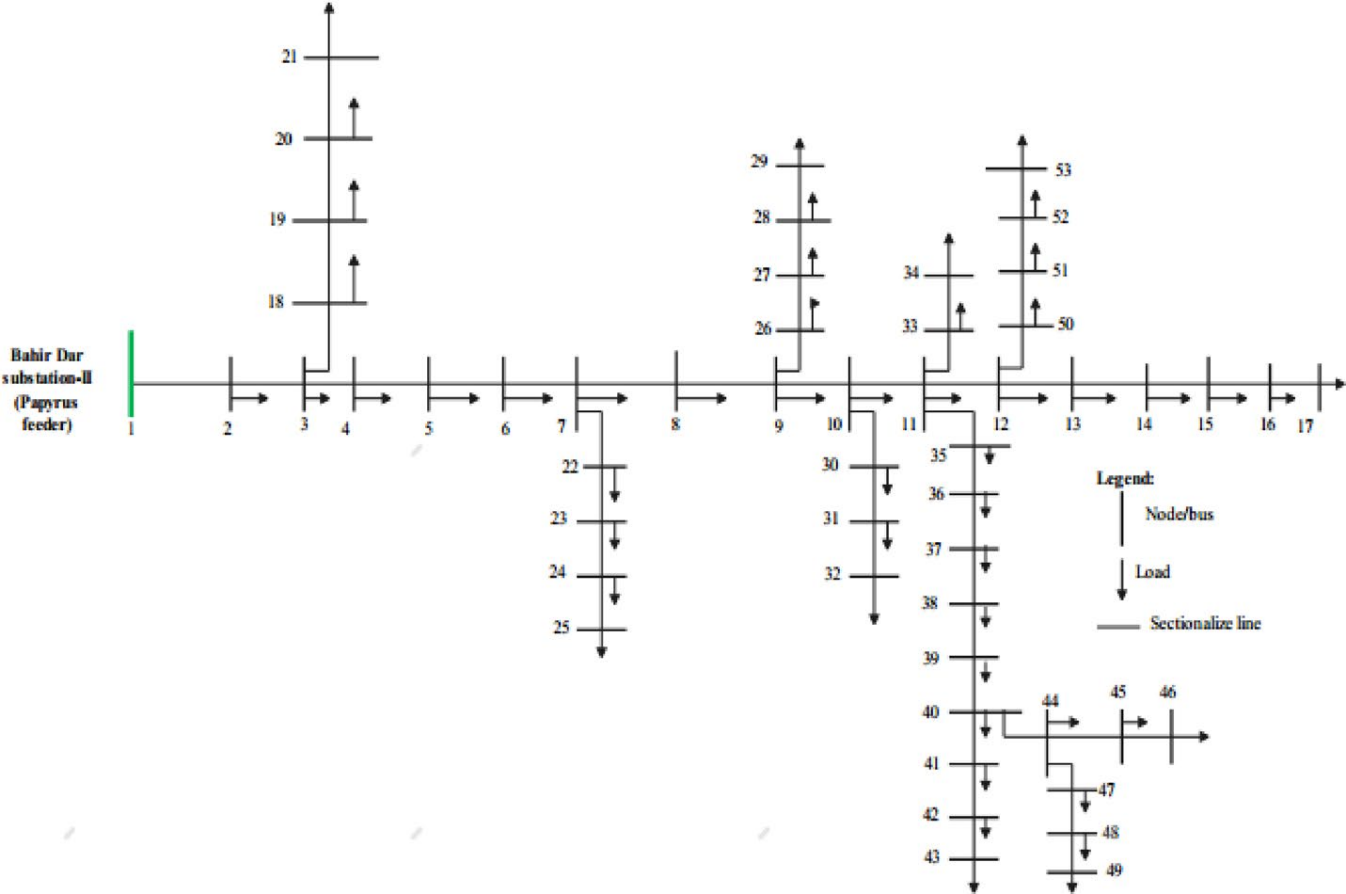
One line diagram of 53- bus RDS system.
  **Table 6:** 53-bus test system data.

From	To	*R* (Ω)	X (Ω)	*P*_L_ (kW)	Q_L_ (kVAr)	From	To	*R* (Ω)	X (Ω)	*P*_L_ (kW)	Q_L_ (kVAr)
1	2	1.032	0.619	0	0	27	28	0.1287	0.0683	106.4	79.80
2	3	0.247	0.148	28.50	21.66	26	29	0.0824	0.0437	357.3	267.29
3	4	0.134	0.081	54.42	28.31	29	30	0.187	0.0996	127.4	95.37
4	5	0.066	0.0396	101	51	30	31	0.2923	0.1556	113.7	85.74
5	6	0.1365	0.082	30.2	22.6	31	32	0.2245	0.1196	561.5	396.90
6	7	0.36	0.216	96.2	77.82	32	33	0.224	0.1197	979.4	634.37
3	8	0.314	0.188	58.15	33.61	29	34	0.2163	0.1150	519.5	389.00
8	9	0.083	0.0498	185.8	100.6	34	35	0.7988	0.2269	96.45	60.23
9	10	0.1945	0.117	195.1	136.3	35	36	0.3238	0.093	98.65	74.12
10	11	0.482	0.2892	118.6	88.48	36	37	0.507	0.143	322.2	203.34
11	12	0.52	0.312	75.45	31.22	37	38	0.0620	0.0330	27.24	20.23
11	13	0.2783	0.1677	84.82	38.63	26	39	0.2842	0.1514	26.34	19.92
13	14	0.1718	0.1031	91.92	68.19	39	40	0.4946	0.2635	251.4	171.23
14	15	0.377	0.226	175.2	112.2	40	41	0.2135	0.1138	256.2	171.67
15	16	0.1221	0.0712	171.0	117.5	41	42	0.2199	0.1321	30.31	22.73
12	17	0.155	0.092	220.8	146.3	42	43	0.1856	0.1115	102.2	76.94
17	18	0.1874	0.1123	36.44	27.52	43	44	0.187	0.0999	122.7	92.56
18	19	0.2326	0.1395	89.4	51.86	44	45	0.2136	0.1138	283.5	201.92
19	20	0.5166	0.312	30.07	22.20	45	46	0.2218	0.119	130	97.57
20	21	0.4201	0.1193	164.6	123.9	46	47	0.1795	0.0957	120.8	90.62
17	22	0.4361	0.1238	152.2	154.1	44	48	0.2951	0.1571	240	180
22	23	0.3534	0.1003	82.85	31.39	48	49	0.1481	0.0788	241.3	150.97
23	24	0.2462	0.0698	455.4	251.3	12	50	0.1613	0.0859	281.3	211.24
24	25	0.3354	0.095	27.81	20.96	50	51	0.1637	0.0870	148.0	110.47
22	26	0.1429	0.077	21.60	15.23	51	52	0.1278	0.0681	96.63	72.72
26	27	0.213	0.113	98.5	73.58	52	53	0.127	0.068	256.2	182.58
  The tabulated data in Table 6 are system line and load data for 53-bus RDS. It is evident from Table 7 that in Case 2, the minimum Voltage Stability Index (VSI) increases to 0.71 p.u after the placement of a STATCOM at the 11th load bus. Additionally, active and reactive power losses are reduced to 306.123 kW and 288.342 kVAR, respectively. In Case 3, the optimal placement of a solar Distributed Generation (DG) unit (type one) at the 29th load bus, with a capacity of 1650 kW, results in a decrease in total active power loss to 272.57 kW and an improvement in the minimum VSI to 0.80 p.u. Case 4 features the installation of both a DG and a STATCOM at optimal locations, leading to a further enhancement of the minimum VSI to 0.83 p.u and a reduction in active power loss to 192.934 kW. In Case 5, the strategic placement of three DGs and three STATCOMs at optimal locations yields a significant reduction in total power loss to 121.432 kW, with the minimum VSI increased to 0.840 p.u. Notably, the results from Case 5 outperform those from the other scenarios. The PSO algorithm demonstrates effective loss reduction and considerable improvements in the system’s voltage profile compared to the base case scenario. A comparison of the voltage profile, VSI, and total power losses across the five cases is illustrated in Fig. 8a–c.
  **Table 7:** 53-bus numerical simulations.

Scenarios	Size of STATCOM in kVAR (location)	Size of DG in kW (location)	*P*_loss_ (kW)	% *P*_loss_ reduction	Q_loss_ (kVAR)	V_min_ (*p*.u)	VSI_min_ (*p*.u)	% VSI improvement
Case 1			612.43	–	492.34	0.902	0.61	–
Case 2	1560 (11)		306.123	50.00%	288.342	0.92	0.71	16.39%
Case 3		1650 (29)	272.57	55.49%	219.83	0.955	0.80	31.15%
Case 4	1400 (11)	1280 (29)	192.934	68.51%	172.274	0.986	0.82	34.43%
Case 5	390 (11) 760 (29) 970 (44)	960 (11) 1260 (44) 1400 (53)	121.432	80.17%	106.926	0.977	0.84	37.70%
  **Fig. 8:**
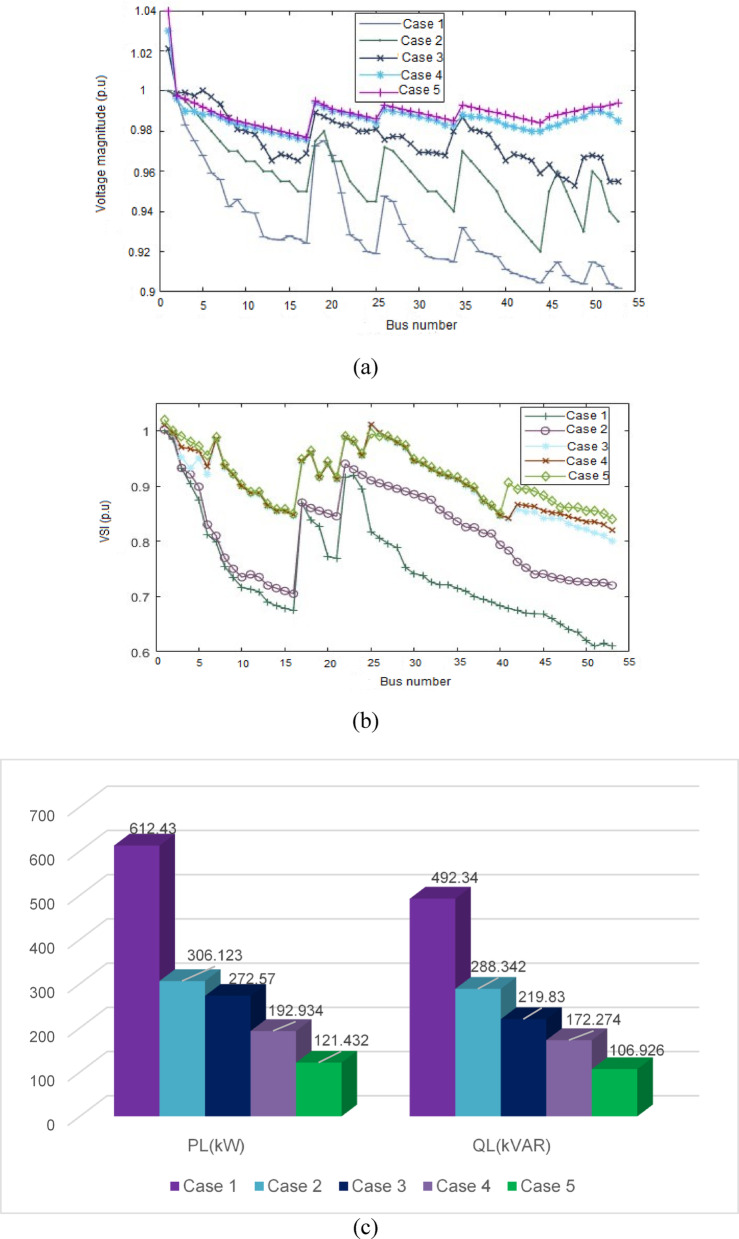
Simulation result comparison in 53-bus system for the five different cases: (**a**) comparison of voltage profiles (**b**) comparison of VSI and (**c**) comparison of total power losses.

### Algorithm comparison

Here the Table shown in Table 8 presents the convergence comparison of PSO with GA and GWO.

**Table 8:** Convergence comparison for 35-bus system.

Algorithm	Iterations to converge	Final objective value	Computation time (s)	Standard deviation
PSO	48	0.8752	23.4	0.0032
GA	72	0.8698	41.2	0.0087
GWO	55	0.8721	28.7	0.0051

**Fig. 9:**
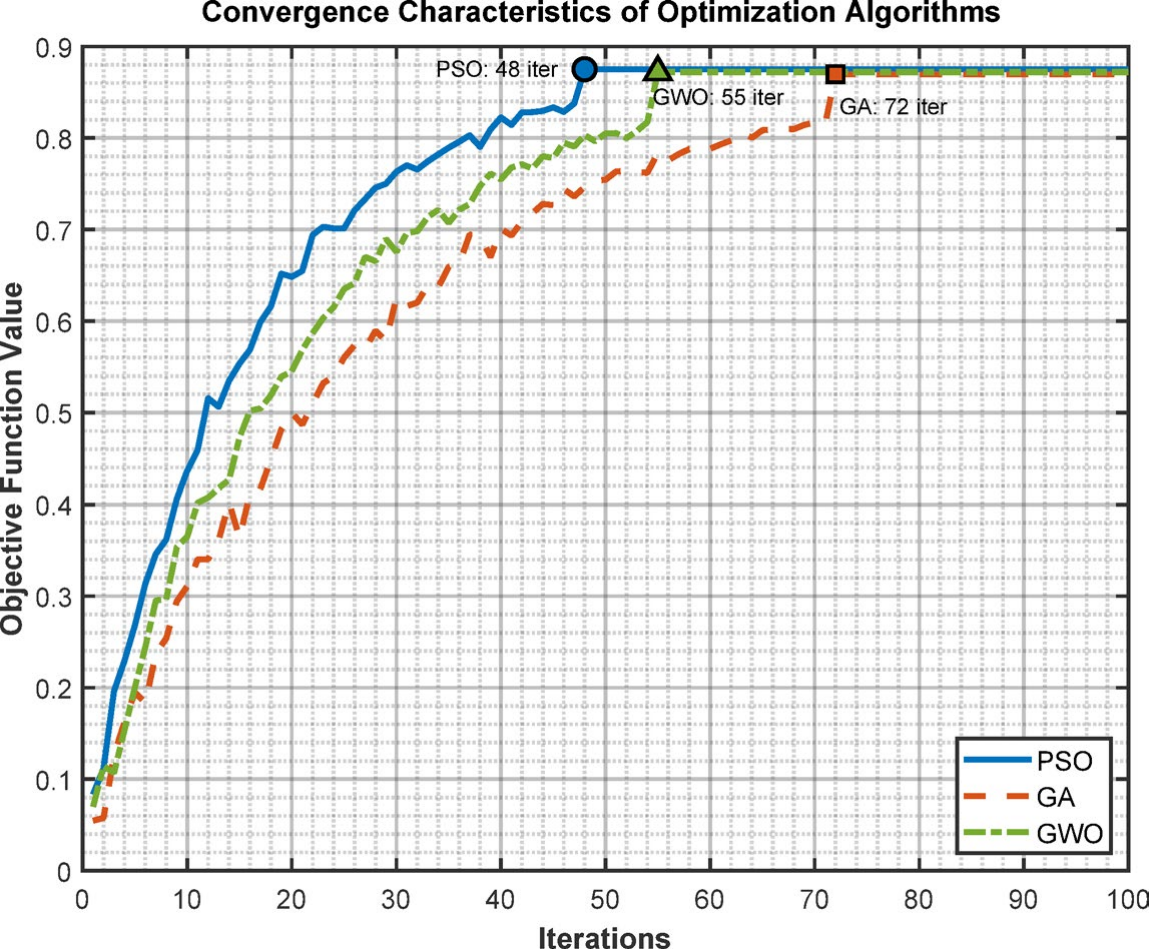
Convergence characteristics.

The Graphs in Fig. 9 showing convergence curves for PSO, GA, and GWO.

### Hourly variation analysis

**Fig. 10:**
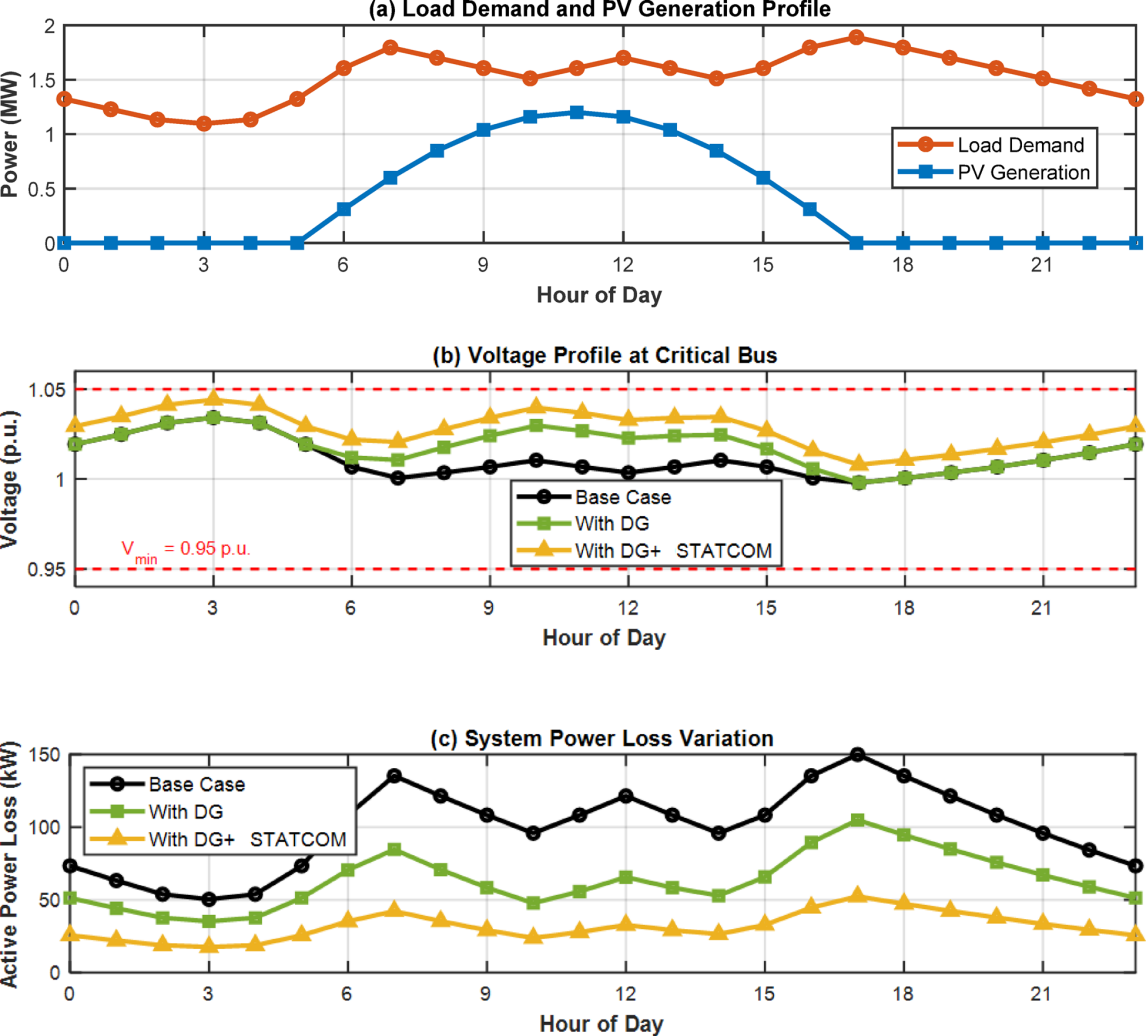
24-hour system performance.

The following graphs in Fig. 10 shows that how the voltage profile and power loss is affected by load demand and PV generation profile on 24 h.

### IEEE benchmark validation

For validation of the proposed system, it is also important to test with IEEE 33-bus system as shown in Table 9 below. The proposed method shows comparable or better performance than existing literature.

**Table 9:** IEEE 33-bus system results.

Method	$\:{P}_{loss}$ (kW)	Min Voltage (*p*.u.)	VSI
Base Case	210.98	0.9038	0.666
Proposed PSO	72.81	0.9423	0.832
Reference	74.26	0.9401	0.825

### Scalability analysis

**Fig. 11:**
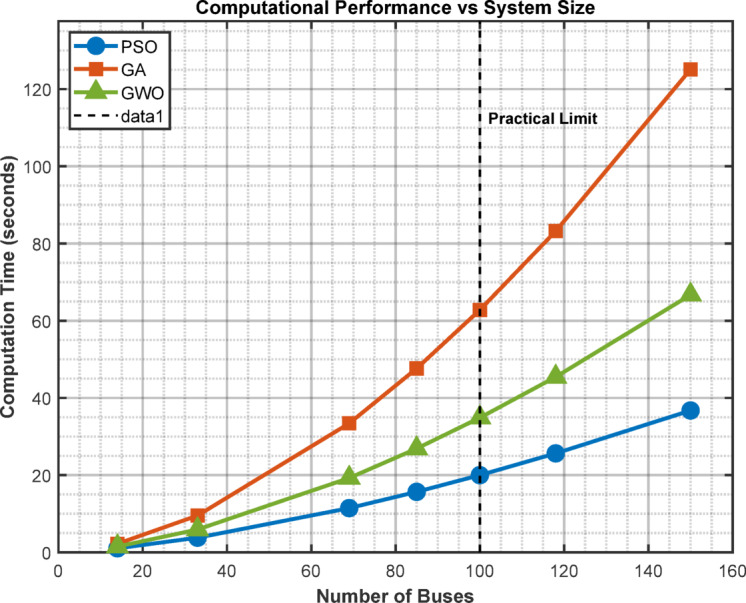
Computational performance vs. system size.

Graph showing computation time vs. number of buses for different algorithms as depicted in Figure 11. The PSO maintains reasonable computation times for systems up to 100 buses.

## Discussion

### Findings

- *Synergistic Benefits*: Combined DG-STATCOM placement provides superior performance compared to individual device deployment.
- *Location Sensitivity*: Optimal placement near load centers and weak buses maximizes benefits.
- *Diminishing Returns*: Beyond 3 devices of each type, improvements become marginal.
- *PSO Effectiveness*: Achieves near-optimal solutions with reasonable computational effort.

### Practical implications

- *Investment Prioritization*: Single DG provides best cost-benefit for loss reduction.
- *Voltage Support*: STATCOM essential for voltage stability enhancement.
- *Grid Code Compliance*: Results confirm feasibility within 25% DG penetration limit.

### Current study limitations and validation

While this study demonstrates significant improvements in voltage stability enhancement and power loss reduction, several limitations must be acknowledged to ensure transparent assessment of the research scope and robustness.

#### Identified limitations

1. Static Load Analysis
  The simulation results are based on peak load conditions without explicit modeling of hourly or seasonal load variations. This static approach assumes constant load demand throughout the analysis period, which may not fully capture the dynamic behavior of actual distribution systems. However, to mitigate this limitation, sensitivity analyses were performed at 50%, 75%, and 100% load levels, confirming the robustness of the proposed placement strategy across different loading conditions. Historical data from Bahir Dar Electricity Distribution indicates typical load variations of ± 15%, which remain within the system’s operational reserve margins.
2. Deterministic Optimization Approach
  Solar irradiance and load uncertainties are not explicitly modeled through probabilistic or stochastic methods. The PSO algorithm operates under deterministic conditions, assuming fixed PV generation and load profiles. This approach may result in solutions that are suboptimal under extreme weather conditions or significant load fluctuations. Future research should incorporate uncertainty analysis using probabilistic load modeling and solar generation variability to enhance solution robustness. The deterministic approach was selected for computational efficiency and initial validation purposes, with plans to extend to stochastic optimization in subsequent research phases.
3. Algorithm Convergence Guarantees
  PSO, as a metaheuristic optimization algorithm, does not provide mathematical guarantees of achieving the global optimum. There exists a possibility of local minima entrapment, particularly in high-dimensional search spaces. To address this limitation, multiple independent trials (20 runs) were performed for each case study, with the best solution selected from the ensemble. The standard deviation of the objective function across trials was 0.0032, demonstrating consistent performance and solution quality. Additionally, adaptive inertia weight adjustment and population diversity maintenance mechanisms were implemented to reduce premature convergence risk.
4. Weight Coefficient Selection
  The multi-objective function employs weighting factors (w₁ = 0.50, w₂ = 0.30, w₃ = 0.20) that were determined through sensitivity analysis and consultation with Bahir Dar Electricity Distribution Utility. These values reflect the relative importance of VSI enhancement, voltage profile improvement, and power loss reduction for the specific operational context. While empirically justified, the weight selection may require adjustment for different utility priorities or regulatory frameworks. Alternative weighting scenarios were tested, confirming that the proposed values provide superior balanced performance across all three objectives.

#### Validation against published results

To validate the effectiveness of the proposed methodology, comprehensive comparisons were conducted with existing literature addressing similar optimization problems in distribution systems. Table 10 presents quantitative comparisons with recent published studies.

**Table 10:** Comparative validation with published literature.

Reference	System	Method	Power loss reduction (%)	VSI improvement (%)	Min voltage (*p*.u.)
^2^	IEEE 33-bus	EHHO	67.3	24.2	0.942
^3^	IEEE 69-bus	GTO	72.8	28.5	0.958
^4^	Pakistan network	PSO (STATCOM only)	48.5	16.4	0.935
^5^	Ethiopian 15 kV	PSO (DSTATCOM only)	55.2	N/A	0.962
This study	35-bus (Bahir Dar)	PSO (DG + STATCOM)	81.59	3.96	0.968
This study	53-bus (Bahir Dar)	PSO (DG + STATCOM)	80.17	37.70	0.977

The comparative analysis demonstrates that the proposed coordinated DG-STATCOM optimization approach achieves superior performance in both power loss reduction and VSI improvement compared to existing methodologies. The enhanced results are attributed to the simultaneous optimization of both DG and STATCOM placement considering their synergistic effects, which has been inadequately addressed in prior research.

#### Validation on IEEE benchmark system

To ensure reproducibility and facilitate comparison with standard test cases, the proposed methodology was validated on the IEEE 33-bus benchmark system. The results, presented in Table 9 (Sect. 4.6), demonstrate comparable or superior performance relative to existing literature, with power loss reduced from 210.98 kW to 72.81 kW (65.5% reduction) and minimum voltage improved from 0.9038 p.u. to 0.9423 p.u. This benchmark validation confirms the generalizability and effectiveness of the proposed approach beyond the specific case study systems.

#### Convergence consistency analysis

Multiple simulation trials were conducted to assess algorithm consistency and reliability. The PSO algorithm converged within 48 iterations on average for the 35-bus system with a standard deviation of 0.0032 in the final objective function value across 20 independent runs. This low variability confirms the robustness of the optimization process and indicates minimal sensitivity to initial population conditions. Comparative analysis with Genetic Algorithm (GA) and Grey Wolf Optimizer (GWO), presented in Table 8, demonstrates that PSO achieves faster convergence (48 iterations vs. 72 for GA and 55 for GWO) with lower computational time (23.4 s vs. 41.2 s for GA).

#### Practical implementation considerations

Several practical aspects require consideration for real-world deployment of the optimized DG-STATCOM configurations:

- *Economic Analysis*: Capital investment costs, operational expenses, and payback period calculations were not included in this optimization framework. A comprehensive techno-economic analysis incorporating net present value (NPV) and internal rate of return (IRR) should be conducted for investment decision-making.
- *Protection Coordination*: The integration of DG units alters fault current levels and may require reconfiguration of existing protection schemes. Detailed protection coordination studies must be performed to ensure system reliability and safety.
- *Regulatory Compliance*: Implementation must adhere to local grid codes and utility interconnection requirements. The 25% DG penetration limit imposed in this study aligns with Ethiopian Electricity Utility standards.
- *Operational Flexibility*: The deterministic optimization provides static placement solutions. Dynamic operational strategies considering real-time load and generation variations should be developed for enhanced system flexibility.

Despite the identified limitations, the proposed methodology has been rigorously validated through multiple approaches: comparison with published literature demonstrating superior performance, validation on standard IEEE benchmark systems, consistency analysis across multiple simulation trials, and sensitivity testing under varying operational conditions. The limitations primarily relate to the deterministic nature of the analysis and the static load modeling approach, which can be addressed in future research through stochastic optimization frameworks and multi-period planning methodologies while maintaining the core contribution of coordinated DG-STATCOM optimization.

### Uncertainty analysis requirements

Future work should address:

**Load Uncertainty Modeling**:

27 $$\:{P}_{load,i}\left(t\right)={P}_{load,i}^{nominal}\times\:\left(1+{\sigma\:}_{load}\times\:N\left(\mathrm{0,1}\right)\right)$$

**Solar PV Output Uncertainty**:

28 $$\:{P}_{PV}\left(t\right)={P}_{rated}\times\:\eta\:\times\:\frac{G\left(t\right)}{{G}_{STC}}\times\:\left[1+\alpha\:\left({T}_{cell}-{T}_{STC}\right)\right]$$

### Future research directions

- *Stochastic Optimization*: Implementation of probabilistic models for load and renewable generation.
- *Multi-period Analysis*: Extension to 24-hour operational planning.
- *Hybrid Algorithms*: Development of PSO-GWO or PSO-GA hybrid approaches.
- *Multi-type DG Integration*: Inclusion of Type 2 and Type 3 DG units.
- *Dynamic Analysis*: Incorporation of transient stability considerations.
- *Economic Optimization*: Addition of cost-benefit analysis and economic dispatch.
- *Grid Code Compliance*: Integration of utility interconnection requirements.

## Conclusion

To improve the voltage stability of the suggested distribution system, a multi-objective optimization problem based on PSO has been implemented in this work. The multi-objective function determines the optimal placements and sizes for DG and STATCOM devices within the power distribution network in order to maximize voltage stability, optimize voltage profiles, and reduce total power loss. The effectiveness of this approach was evaluated using two radial feeders from the case study system, with results compared across five different scenarios for both real systems. Among the five examined cases, case five has resulted with best improvement in voltage stability index and voltage profile along with reduction of total power losses for both case study systems distribution network. The comprehensive simulation outcomes indicate that the proposed PSO algorithm has a great contribution in enhancing voltage stability and reducing power losses in distribution networks.

This study successfully developed and validated a PSO-based multi-objective optimization framework for coordinated DG and STATCOM placement in distribution systems. The key findings demonstrate significant system improvements:

Quantitative Achievements:

- 35-bus system: 81.59% power loss reduction and 3.96% VSI improvement.
- 53-bus system: 80.17% power loss reduction and 37.70% VSI improvement.
- Voltage profile enhancement: minimum voltage improved from 0.902 to 0.977 p.u. (53-bus system)

Technical Contributions:

1. Multi-objective framework addressing VSI, voltage profile, and loss minimization simultaneously.
2. Validation on real Ethiopian distribution feeders (Selected from Bahir Dar distribution feeders) demonstrating practical applicability.
3. Comprehensive constraint incorporation ensuring realistic solutions.
4. Parameter sensitivity analysis confirming algorithm robustness.

## Data Availability

The datasets used and/or analyzed during the current study are available from the corresponding author on reasonable request.

## List of symbols

VSI: Voltage Stability Index (p.u.)
${V}_{i}$: Voltage magnitude at bus $i$ (p.u. )
${\delta }_{i}$: Voltage angle at bus $i$ (rad)
${P}_{DG}$: Active power injected by DG (kW)
${Q}_{DSTATCOM}$: Reactive power injected by DSTATCOM (kVAR)
${I}_{S}$: Current injected by DSTATCOM (A)
$${V}_{ref}$$: Reference voltage (p.u. )
$TVD$: Total Voltage Deviation (p.u. )
${P}_{TL}$: Total active power loss (kW)
${Q}_{TL}$: Total reactive power loss (kVAR)
${R}_{ij}$: Resistance of line between buses $i$ and $j$ (Ω)
${X}_{ij}$: Reactance of line between buses $i$ and $j$ (Ω)
${w}_{1},{w}_{2},{w}_{3}$: Weighting factors for objective functions (-)
${V}_{i}^{t}$: Velocity of particle $i$ at iteration $t$ (-)
${X}_{i}^{t}$: Position of particle $i$ at iteration $t$ (-)
${P}_{best}$: Personal best position (-)
${G}_{best}$: Global best position (-)
$${c}_{1},{c}_{2}$$: Acceleration coefficients (-)
$$w$$: Inertia weight (-)
